# High-energy guanine nucleotides as a signal capable of linking growth to cellular energy status via the control of gene transcription

**DOI:** 10.1007/s00294-019-00963-1

**Published:** 2019-04-01

**Authors:** Andy Hesketh, Stephen G. Oliver

**Affiliations:** 1 School of Pharmacy and Biomolecular Sciences, University of Brighton, Huxley Building, Lewes Road, Brighton, BN2 4GJ UK; 2 Cambridge Systems Biology Centre and Department of Biochemistry, University of Cambridge, Cambridge, CB2 1GA UK

**Keywords:** Guanylate energy charge, GTP, Regulation, Metabolism

## Abstract

This mini-review considers the idea that guanylate nucleotide energy charge acts as an integrative signal for the regulation of gene expression in eukaryotic cells and discusses possible routes for that signal’s transduction. Gene expression is intimately linked with cell nutrition and diverse signaling systems serve to coordinate the synthesis of proteins required for growth and proliferation with the prevailing cellular nutritional status. Using short pathways for the inducible and futile consumption of ATP or GTP in engineered cells of *Saccharomyces cerevisiae,* we have recently shown that GTP levels can also play a role in determining how genes act to respond to changes in cellular energy supply. This review aims to interpret the importance of GTP as an integrative signal in the context of an increasing body of evidence indicating the spatio-temporal complexity of cellular de novo purine nucleotide biosynthesis.

## Introduction

**Fig. 1:**
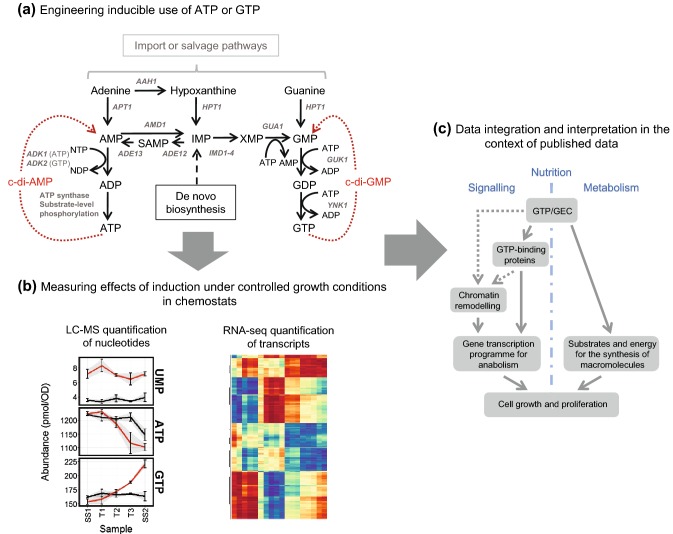
Exploring the effects of increased use of the energy stored in ATP or GTP on gene transcription in the budding yeast *Saccharomyces cerevisiae* (Hesketh et al. 2019). The inducible heterologous expression of bacterial enzymes forms futile shunt pathways to AMP or GMP (**a**) capable of influencing intracellular nucleotide composition and gene transcription (**b**). Data interpretation alongside published information on the correlation of anabolic gene transcription with nucleosome remodeling (Machné and Murray 2012; Nocetti and Whitehouse 2016) suggests GTP/GEC as an integrative signal linking growth to energy status (**c**)

Life requires energy, and the proliferation of life even more so. The common energy currency in living cells is ATP, generated from oxidative and substrate-level phosphorylation and consumed to drive the fundamental processes of DNA maintenance, synthesis and replication, the expression of genes to produce RNA and proteins, and the transport and movement of chemicals and macromolecules. Of these, gene expression—chromatin remodeling, transcription initiation, transcription elongation, mRNA splicing, and translation—accounts for the majority of cellular energy demand, with ~ 75% frequently offered as an estimate (Lane and Martin 2010). Whether gene transcription is responsive to prevailing cellular energetic conditions is, therefore, of fundamental interest. We recently sought to answer this question by developing methods for manipulating metabolic demand for ATP and GTP in a yeast model system, measuring responses in both cellular energy status and the transcriptome (Fig. 1) (Hesketh et al. 2019).

What is meant by cellular energy status, and what is the significance of GTP? A useful way of representing energy status is in terms of the cellular adenylate energy charge (AEC)—defined as the relative concentrations of all three phosphorylated adenosine nucleotides [ATP] + 0.5[ADP]/[ATP] + [ADP] + [AMP] (Atkinson and Walton 1967). The concept of AEC as an integrator capable of signaling changes in the regulation of cell proliferative processes is well established (Hardie et al. 2016; Hoxhaj et al. 2017). The closely related high-energy purine nucleotide in cells, GTP, is usually overlooked in this context because it: (1) is not the major initial product of cellular energy generation (2) is less abundant than ATP in cells, and (3) can readily be produced from ATP by phosphotransfer to GDP. GTP is, however, the immediate source of energy for the highly demanding process of protein synthesis, where two molecules of GTP are consumed for each amino acid incorporated into the growing polypeptide chain. It is also required for the assembly and functioning of the cell cytoskeleton and endoplasmic reticulum and is, in addition, central to the signaling functions of intracellular G-protein switches. The ability of cells to modulate the expression of their genes in response to changes in both guanylate and adenylate energy charge would, therefore, make good physiological sense. In particular, the evolution of a role for GEC as an integrative signal would provide a direct link between energy metabolism and protein synthesis.

## GTP/GEC levels can modulate gene transcription in yeast

To explore the effects of increasing the metabolic use of the energy stored in ATP or GTP on gene transcription in the budding yeast *Saccharomyces cerevisiae*, strains were engineered for the inducible futile conversion of two NTP molecules to two lower energy NMP molecules, via non-native cyclic-di-NMP intermediates (Fig. 1a) (Hesketh et al. 2019). In order to ensure well-defined physiological conditions, our experiments were performed on yeast cells grown in continuous culture in chemostats (Fig. 1b). Cultivation in chemostats, where cells grow at a fixed rate in constant nutritional conditions, was used to control for confounding effects of any changes in growth rate or external nutrient supply during induction. Surprisingly, the resulting changes in transcription we observed were most consistently associated with changes in GTP and GEC levels, although the reprogramming in gene expression during glucose repression was sensitive to adenine nucleotide levels. During steady-state growth using the fermentable carbon source glucose, the futile consumption of ATP led to a decrease in intracellular ATP concentration but an increase in GTP and GEC. Expression of transcripts encoding proteins involved in ribosome biogenesis, and those previously reported to be controlled by promoters subject to SWI/SNF-dependent chromatin remodeling (Amariei et al. 2013; Machné and Murray 2012; Nocetti and Whitehouse 2016), was correlated with these nucleotide pool changes.

## How might a GTP/GEC signal be transduced?

In prokaryotic systems GTP levels can be directly sensed via influencing the selection of transcription start sites by RNA polymerase (Krásný et al. 2008) or though allosteric effects on the binding activities of transcriptional regulators (Brinsmade 2017; Ratnayake-Lecamwasam et al. 2001). There are also examples of eukaryotic genes whose transcription can be controlled by the initiating nucleotide. While a notable example in yeast is the influence of GTP on the transcription of *IMD4* (encoding inosine monophosphate dehydrogenase [IMPDH], a key enzyme in guanine nucleotide biosynthesis) in *S. cerevisiae* (Kuehner and Brow 2008), there is no evidence that this is a widespread occurrence. An influence on the activity of signaling pathways regulated by GTPases is a more likely hypothesis. Evidence for an influence of guanine nucleotide pools on the level of active, GTP-bound, Ras2p has previously been reported (Besozzi et al. 2012; Cazzaniga et al. 2008; Pescini et al. 2012), and the signaling activity of mTORC1 has similarly been shown to be responsive to guanine nucleotide availability (in addition to adenine nucleotides) through alterations in the level of the active, GTP-bound Rheb-GTPase (Emmanuel et al. 2017). While yeast TORC1 lacks a direct Rheb homolog, and the timeliness of the effect of GTP on Rheb-GTPase is under debate (Hoxhaj et al. 2017), control of the activity of TOR-complex signaling by GTPase switches is a conserved feature of signal transduction between yeast and mammals. An increase in the activity of either the Ras/PKA or TORC1 pathways in yeast through elevated GTP levels would be expected to up-regulate transcription of genes associated with growth processes. Alternative protein targets for sensing GTP cannot, however, be excluded. A reverse genetics approach identified a GTP-binding domain in the lipid kinase PI5P4Kβ which functions to convert GTP concentration cues into phosphatidylinositol 5-phosphate (PI(5)P) second messenger signaling for the control of metabolism and tumorigenesis (Sumita et al. 2016; Takeuchi et al. 2016).

The unusual dynamic spatial organization of the enzymes required for purine biosynthesis into cellular macrostructures, filamentous cytoophidia (Aughey and Liu 2015; Chang et al. 2015; Keppeke et al. 2015) and purinosomes (An et al. 2008; French et al. 2016; Pedley and Benkovic 2017), may also offer a potential route for the control of gene expression by GTP in eukaryotes. The IMPDH enzyme, which controls a rate-limiting step for guanine nucleotide synthesis, has been shown to moonlight as a cell-cycle-regulated transcription factor in *Drosophila* cells, mediating the repression of histone genes and E2F, a key driver of cell proliferation (Kozhevnikova et al. 2012). *E. coli* IMPDH was also shown to exhibit the same sequence-specific DNA-binding activity as the *Drosophila* enzyme, suggesting that moonlighting as a transcriptional regulator may be a broadly conserved function of this enzyme (Kozhevnikova et al. 2012). Interestingly, IMPDH in mammalian cells has also been shown to undergo assembly into cytoplasmic filaments, known as cytoophidia, during periods of rapid cell proliferation (Chang et al. 2015; Keppeke et al. 2018), a process which is promoted by intracellular IMP accumulation and antagonized by elevated levels of guanine nucleotides (Keppeke et al. 2018). While believed to be a mechanism for controlling metabolic flux through the biosynthesis pathway, reversible aggregation could also be expected to affect its function as a transcriptional regulator by influencing transport into the nucleus.

Upstream of IMPDH, many of the enzymes required for de novo IMP biosynthesis have been observed to dynamically assemble and disassemble into a multi-enzyme cytoplasmic macrostructure termed the purinosome (Pedley and Benkovic 2017). The transient nature of purinosomes has made them challenging to characterize and study, but a consensus is emerging in which it is believed that purinosome formation enhances IMP synthesis and is spatially focused around mitochondria and microtubules (Chan et al. 2018; French et al. 2016; Zhao et al. 2015). The proximity of mitochondrial ATP production, GTP-fueled microtubule formation, and the energy intensive process of de novo purine biosynthesis is intriguing and offers opportunities for functional harmonization. Whether this is just limited to a sharing and channeling of common nucleotide metabolites or extends to include regulatory interactions is an interesting question. Retrograde signaling communication between mitochondria and the nucleus coordinates mitochondrial protein synthesis and communicates mitochondrial functional status, triggering compensatory responses in nuclear gene expression. On a global level, cell-to-cell differences in mitochondrial content can account for much of the variability in average rates of cellular transcription observed in populations of identical eukaryotic cells, with an increased mitochondrial mass correlating with increased chromatin activation and RNA polymerase II activity (Guantes et al. 2015; das Neves et al. 2010). ATP is thought to be the prime driver behind these effects, but a contribution from GTP has yet to be considered, not least because GTP levels tend to shadow those of ATP. As part of this complexity, the proliferation of mitochondria by membrane fission has recently been shown to be driven by GTP, produced at the site of action from ATP by a member of the division machinery complex, DYNAMO1 (Imoto et al. 2018). An homologous protein DYNAMO2 has recently been proposed as a regulator of global GTP levels during the cell cycle of the red alga *Cyanidioschyzon merolae* (Imoto et al. 2019).

## Puzzles and prospects

Testing the hypotheses discussed above concerning the mechanisms by which high-energy guanine nucleotide status modulates gene transcription will require multidisciplinary investigations using the latest techniques in molecular biology and fluorescence microscopy. How induction of the ATP- or GTP-consuming pathways affects formation of IMPDH filaments and purinosomes, and how the abundance of activated GTPase switch proteins is influenced are key questions yet to be answered. The synthesis and use of high-energy adenine and guanine nucleotides are intimately linked (see Fig. 1) and obtaining a clear view of the control exerted by GTP from amongst the shadow cast by ATP will be challenging. Inhibitors of IMPDH activity have been used to good effect for specifically lowering GTP levels relative to ATP (see Emmanuel et al. 2017; Hoxhaj et al. 2017) but are of limited use for modulating GEC, since they also inhibit the production of GMP and GDP. Specific inhibition of the conversion of GDP to GTP would be desirable, but has yet to be achieved.

The success of future work will depend on the ability to cleanly dissect the in vivo effects of GTP/GEC from those of ATP/AEC, using tools to manipulate the levels of these closely related nucleotides independently from one another. Recent in vitro studies analyzing the filamentation state and activity of human IMPDH enzymes indicate differential allosteric responses to adenine and guanine nucleotides such that IMPDH cytoophidia formation facilitates the accumulation of high levels of guanine nucleotides when the cell requires them (Fernández-Justel et al. 2019). A similar mechanism in yeast may explain a surprising observation in our own recent study, where induction of the ATP-consuming pathway produced a net increase in GEC and GTP concomitant with a decrease in the concentration of ATP and a stable AEC. Genetic approaches to understand and develop this differential activity may, therefore, provide a useful way forward and provide conclusive evidence of the key integrative role of GEC or GTP in the economy of the eukaryotic cell.
